# Can hypoxic exercise retard cellular senescence? A narrative review

**DOI:** 10.1186/s11556-024-00352-9

**Published:** 2024-11-13

**Authors:** Tinghuai Huang, Charlotte Tsang, Jianwei Huang

**Affiliations:** 1 Department of Rehabilitation Sciences, The Hong Kong Polytechnic University, Hong Kong, China; 2 Guangzhou Medical University, Guangzhou, Guangdong China; 3 The Fifth Affiliated Hospital of Guangzhou Medical University, Guangdong, China

**Keywords:** Hypoxia, Physical exercise, Senescence, Altitude

## Abstract

**Background:**

Senescent cells are defined as normal cells that have undergone irreversible division arrest due to various factors. These cells have been found to play a pivotal role in aging and the development of chronic diseases. Numerous studies demonstrated that physical exercise is effective in anti-aging and anti-chronic diseases. Furthermore, the combination of exercise and hypoxia has been shown to optimize the stimulus of oxygen deprivation and extend cellular lifespan.

**Objective:**

This narrative review offers an exhaustive analysis of existing literature studying the effect of hypoxic exercise on cellular senescence under various conditions.

**Methods:**

Four electronic databases underwent title and abstract screening to summarize the effect of hypoxic exercise on cellular senescence under various conditions. Papers were deemed eligible if they examined the effect of hypoxic exercise on cellular senescence in full-text, peer-reviewed journals and published in English. The final search was carried out on May 4, 2024. Studied were excluded if they: (a) did not involve the utilization of hypoxic exercise as a sole intervention or a contributing factor; (b) did not investigate cellular senescence; (c) lacked sufficient information regarding the study design and findings. A total of 2033 articles were obtained from four databases. However, only 11 articles were deemed to meet eligibility criteria after thoroughly examining titles, abstracts, and full-text content. Authorship, publication year, details of the experimental subject, types of exercise, training protocols, organ, tissue or cell, markers of senescent cells examined, and their responses elicited by exercise were diligently recorded.

**Results:**

This review identified 11 articles for data extraction. The sample sizes varied across a spectrum of complexity, ranging from 4 to 60 (Median=20). The studied population encompassed different healthy cohorts, which comprised sedentary males (n=6), trained males (*n*=2), mountain climbers (*n*=1), and older adults (n=2). Included studies preferred using bicycle ergometers (72.7%, *n*=8) as the exercise modality and 10 studies (90.9%) utilized hypoxia chambers to mimic a normobaric hypoxia environment. Four studies (36.4%) opted to utilize hypoxia chambers to mimic an altitude of 2733 and 4460 m. Additionally, 54.5% of studies (*n*=6) specifically investigated the effect of hypoxic exercise on lymphocytes, commonly utilizing CD28 (*n*=3) and CD57 (*n*=3) as markers of cellular senescence. Four studies (33.3%) examined the impact of hypoxic exercise on erythrocytes using CD47 as the marker for detecting senescent cells.

**Conclusion:**

These data support the notion that hypoxic exercise can retard cellular senescence of specific cells. In the future, standardization on the type of hypoxic exercise and markers of cellular senescence will be essential. Additionally, greater attention should be given to female populations and patients with different disease states. Lastly, further studies of the optimal form and dosage of exercise and the underlying cellular mechanisms are warranted.

**Trial registration:**

PROSPERO, identifier CRD42023431601.

## Introduction

Senescent cells are characterized as normal cells that have undergone irreversible division arrest owing to a variety of factors, including telomere shortening, metabolic, DNA damage, inflammation and mitochondrial dysfunction [10, 15, 30]. Senescent cells can secrete a complex paracrine response characterized by releasing proinflammatory cytokines, proteases, growth factors, chemokines and so on [19, 64]. The senescence-associated secretory phenotype (SASP) plays a significant role in causing local and systemic dysfunction both during the aging process and in various diseases [3, 12, 41, 45, 54, 63]. The selective elimination of senescent cells has been shown to provide notable benefits in specific tissues such as adipose tissue and skeletal muscle [3, 4]. Nevertheless, recent studies have revealed the beneficial roles of senescent cells, in preventing the propagation of pre-malignant cells, which provide a critical barrier against tumorigenesis [17]. These findings prompt further investigation into the varying functions of senescent cells across different tissues.

Hypoxia is characterized by decreased oxygen availability at blood, lungs, or tissue levels. While hypoxia is commonly acknowledged as an essential pathophysiological mechanism in various diseases, such as sleep apnea [35], there are arguments indicating hypoxia can potentially delay different forms of premature senescence in cells cultured under normal oxygen conditions. It has been suggested that extreme hypoxic conditions (0.2% oxygen) can even prevent the induction of senescence [7, 32, 36].

Physical exercise is universally recognized as a safe, potent, and cost-efficient “medicine” for addressing a range of age-related diseases. Hypoxic exercise refers to a type of physical activity that is performed under reduced oxygen conditions, simulating varying levels of altitude either by altering the density of oxygen within the area or traveling to locations of varying altitudes [20]. Multiple arguments indicate that hypoxic exercise can potentially improve cellular circulation and prolong cellular lifespan [16, 49]. Meanwhile, a wealth of evidence indicates that the combination of exercise and hypoxia can optimize the stimulus of oxygen deprivation, resulting in enhanced cellular circulation and potentially prolonged cellular lifespan, surpassing those of normobaric exercise [16, 49, 55].

However, to our knowledge, a clear consensus on the impact of hypoxic exercise on the senescence of various cell types is lacking. Hence, our objective was to examine the impact of hypoxic exercise on senescent cells to support the hypothesis that hypoxic exercise can retard cellular senescence. The findings of the study would deepen our understanding of how hypoxic exercise affects cellular senescence in various tissues. These results could inspire individuals to their physical activity level and open doors to develop personalized exercise plans that cater to the needs of the aging population and individuals with age-related diseases.

### Review methodology

This review followed the guidelines provided in the PRISMA statement [37]. An electronic database search was performed on titles and abstracts using Scopus, Cochrane, PubMed and Web of Science. The final search was carried out on May 4, 2024. Search query, based on the PICO strategy [37]: cellular senescence, hypoxia and exercise, is summarized in Table 1. This review was registered in PROSPERO and the identifier is CRD42023431601. Table 1 shows the final search query based on the PICO strategy [37].

**Table 1:** Search string used for each database

Database	Search string	Records
Scopus	#1: (TITLE-ABS-KEY(senescent) OR TITLE-ABS-KEY(senescence) OR TITLE-ABS-KEY(senescence, AND cellular) OR TITLE-ABS-KEY(cell AND senescence) OR TITLE-ABS-KEY(senescence, AND cell) OR TITLE-ABS-KEY(cell AND aging) OR TITLE-ABS-KEY(cellular AND ageing) OR TITLE-ABS-KEY(ageing, AND cellular) OR TITLE-ABS-KEY(aging, AND cell) OR TITLE-ABS-KEY(senescence, AND replicative) OR TITLE-ABS-KEY(cellular AND aging) OR TITLE-ABS-KEY(aging AND cellular) OR TITLE-ABS-KEY(replicative AND senescence) OR TITLE-ABS-KEY(cell AND ageing) OR TITLE-ABS-KEY(ageing, AND cell) OR TITLE-ABS-KEY(senescence-associated AND secretory AND phenotype) OR TITLE-ABS-KEY(phenotype, AND senescence-associated AND secretory) OR TITLE-ABS-KEY(secretory AND phenotype, AND senescence-associated) OR TITLE-ABS-KEY(senescence AND associated AND secretory AND phenotype) OR TITLE-ABS-KEY(sasp))	293
	#2: (TITLE-ABS-KEY (hypoxia) OR TITLE-ABS-KEY (hypobaric) OR TITLE-ABS-KEY (normobaric) OR TITLE-ABS-KEY (high AND altitude))	
	#3: (TITLE-ABS-KEY (exercise) OR TITLE-ABS-KEY (training) OR TITLE-ABS-KEY (sport) OR TITLE-ABS-KEY (physical AND activity) OR TITLE-ABS-KEY (strength))	
	#1 AND #2 AND #3	
Cochrane	#1: (senescent):ti,ab,kw OR (senescence):ti,ab,kw OR (senescence, cellular):ti,ab,kw OR (cell senescence):ti,ab,kw OR (senescence, cell):ti,ab,kw OR (cell ageing):ti,ab,kw OR (cellular ageing):ti,ab,kw OR (ageing cellular):ti,ab,kw OR (aging, cell):ti,ab,kw OR (senescence, replicative):ti,ab,kw OR (cellular aging):ti,ab,kw OR (aging, cellular):ti,ab,kw OR (replicative senescence):ti,ab,kw OR (cell ageing):ti,ab,kw OR (ageing, cell):ti,ab,kw OR (senescence-associated secretory phenotype):ti,ab,kw OR (phenotype, senescence-associated secretory):ti,ab,kw OR (secretory phenotype, senescence-associated):ti,ab,kw OR (senesence associated secretory phenotype):ti,ab,kw OR (SASP):ti,ab,kw	12
	#2: (hypoxia):ti,ab,kw OR (hypobaric):ti,ab,kw OR (normobaric):ti,ab,kw OR (high altitude):ti,ab,kw	
	#3 (exercise):ti,ab,kw OR (training):ti,ab,kw OR (sport):ti,ab,kw OR (physical activity):ti,ab,kw OR (strength):ti,ab,kw	
	#1 AND #2 AND #3	
PubMed	#1 (((((((((((((((((((senescent) OR (senescence)) OR (senescence, cellular)) OR (cell senescence)) OR (senescence, cell)) OR (cell aging)) OR (cellular ageing)) OR (ageing, cellular)) OR (aging, cell)) OR (senescence, replicative)) OR (cellular aging)) OR (aging, cellular)) OR (replicative senescence)) OR (cell ageing)) OR (ageing, cell)) OR (senescence-associated secretory phenotype)) OR (phenotype, senescence-associated secretory)) OR (secretory phenotype, senescence-Associated)) OR (senescence associated secretory phenotype)) OR (SASP)	572
	#2 (((hypoxia) OR (hypobaric)) OR (normobaric)) OR (high altitude)	
	#3 ((((exercise) OR (training)) OR (sport)) OR (physical activity)) OR (strength)	
	#1 AND #2 AND #3	
Web of Science	#1: (((((((((((((((((((((((((((((((((((((((TS=(senescent)) OR TI=(senescent)) OR TS=(senescence)) OR TI=(senescence)) OR TS=(senescence, cellular)) OR TI=(senescence, cellular)) OR TS=(cell senescence)) OR TI=(cell senescence)) OR TS=(senescence, cell)) OR TI=(senescence, cell)) OR TS=(cell aging)) OR TI=(cell aging)) OR TS=(cellular ageing)) OR TI=(cellular ageing)) OR TS=(ageing, cellular)) OR TI=(ageing, cellular)) OR TS=(aging, cell)) OR TI=(aging, cell)) OR TS=(senescence, replicative)) OR TI=(senescence, replicative)) OR TS=(cellular aging)) OR TI=(cellular aging)) OR TS=(aging, cellular)) OR TI=(aging, cellular)) OR TS=(replicative senescence)) OR TI=(replicative senescence)) OR TS=(cell ageing)) OR TI=(cell ageing)) OR TS=(ageing, cell)) OR TI=(ageing, cell)) OR TS=(senescence-associated secretory phenotype)) OR TI=(senescence-associated secretory phenotype)) OR TS=(phenotype, senescence-associated secretory)) OR TI=(phenotype, senescence-associated secretory)) OR TS=(secretory phenotype, senescence-associated)) OR TI=(secretory phenotype, senescence-associated)) OR TS=(senescence associated secretory phenotype)) OR TI=(senescence associated secretory phenotype)) OR TS=(SASP)) OR TI=(SASP)	1156
	#2: (((((((TS=(hypoxia)) OR TI=(hypoxia)) OR TS=(hypobaric)) OR TI=(hypobaric)) OR TS=(normobaric)) OR TI=(normobaric)) OR TS=(high altitude)) OR TI=(high altitude)	
	#3: (((((((((TS=(exercise)) OR TI=(exercise)) OR TS=(training)) OR TI=(training)) OR TS=(sport)) OR TI=(sport)) OR TS=(physical activity)) OR TI=(physical activity)) OR TS=(strength)) OR TI=(strength)	
	#1 AND #2 AND #3	

A series of specific inclusion criteria were applied to screen the articles: (a) original study; (b) scholarly articles; (c) English-written articles; (d) articles with full-text accessibility; (e) hypoxic exercise was implemented either in isolation as a single intervention or in conjunction with other approaches; (f) Investigate cellular senescence in various organs or tissues. After removing duplicated findings, the remaining articles underwent a meticulous evaluation for relevance based on their titles and abstracts, and they were thoroughly reviewed in full. Studied were excluded: (a) did not involve the utilization of hypoxic exercise as a sole intervention or a contributing factor; (b) did not investigate cellular senescence; (c) lacked sufficient information regarding the study design and findings. Two separate reviewers meticulously assessed the titles and abstracts of the articles obtained from electronic databases, following predefined eligibility criteria. Then, the reviewers conducted a detailed examination of the full text of each chosen article to confirm their eligibility. Reviewers worked collaboratively to reach a consensus on any discrepancies. Extracted variables from the selected research articles were categorized and tabulated following predefined criteria. Authorship, publication year, details of the experimental subject, types of exercise, training protocols, organ, tissue or cell, markers of senescent cells examined and their responses elicited by exercise were diligently recorded.

## Result

**Fig. 1:**
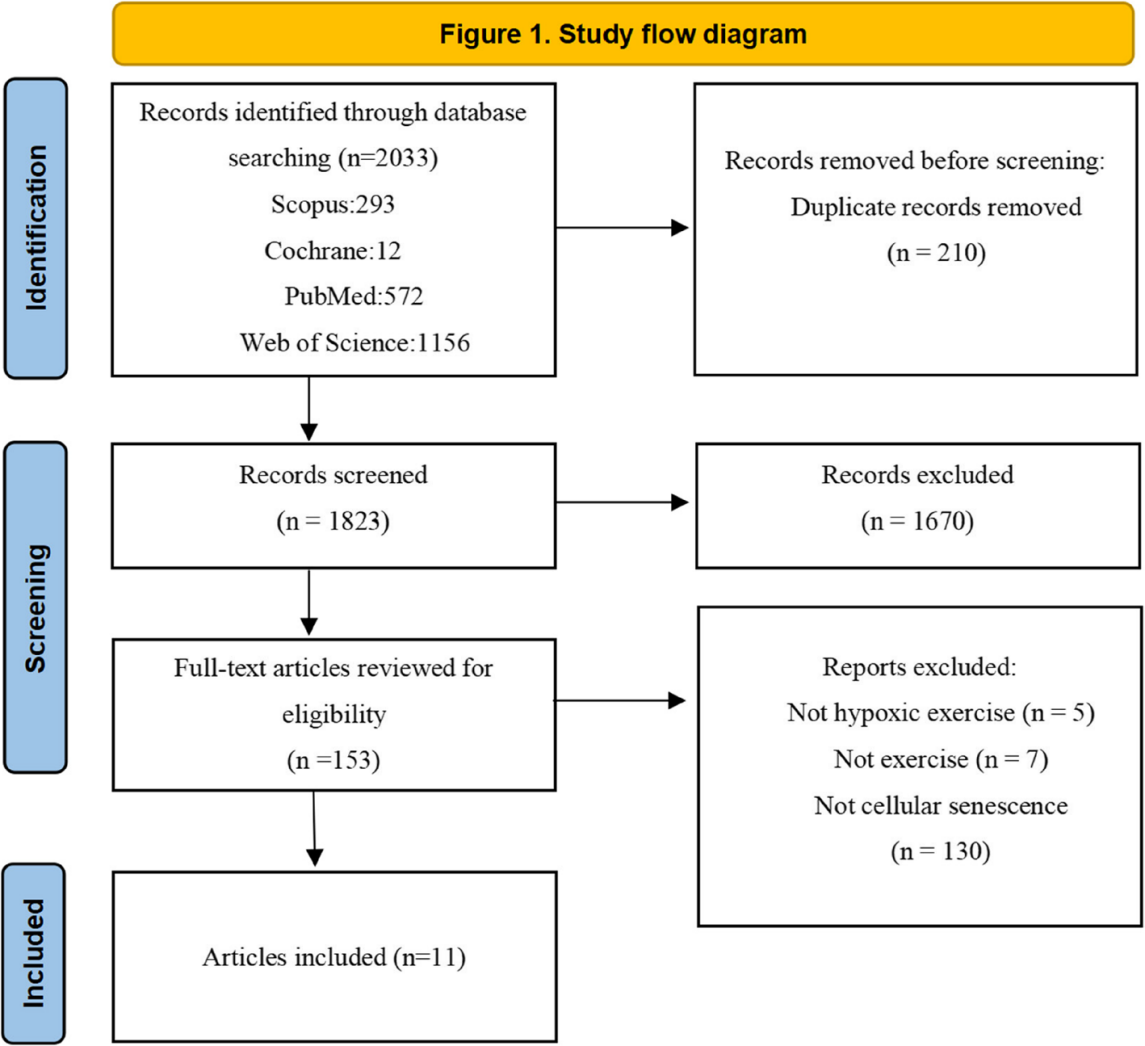
Study flow diagram

The database search initially retrieved 2033 articles. After removing 210 duplicates, 1823 papers remained for screening. 1670 records were excluded based on the predefined criteria. Following a full-text review of the remaining 153 articles, 11 were shortlisted in this review. A comprehensive overview of the selection process is provided in Figure 1.

This review investigated the relationship between hypoxic exercise and senescent cell activity (Table 2). The number of subjects involved in examining the effect of hypoxic exercise varied across a spectrum of complexity, with sample sizes ranging from 4 to 60 (Median=20). The studied population encompassed different healthy cohorts, which comprised sedentary males (*n*=6) (Table 3), trained males (*n*=2), mountain climbers (*n*=1), and older adults (*n*=2).

**Table 2:** Summary of effect of hypoxic exercise on senescence cells

Author (Year of publication)	Studied population	Study design	Exercise	Exercise protocol	Organ or tissue examined	Altitude attained/mimicked	Use of normobaric hypoxia chambers^a^	Marker of SCs	Retard SCs
Harmsen, Nebe et al. [27]	4 healthy trained young males (23±2 years)	Single-group study	Bicycle ergometer	Performed warm-up at 70 W for 10 min and rested for 5 min. Started at 100 W, increased 30 W/3min, maintained at 70-80 rpm, aborted when PR<60 rpm.	Circulating endothelial progenitor cells	4000 m	Y	SA-β-gal	Y
Carin, Deglicourt et al. [11]	9 endurance-trained male cyclists (30.0±8.5 years)	Crossover study	Bicycle ergometer	Performed warm-up at 90 W for 3min. Started at 90 W, increased 30 W/min, maintained at 70-90 rpm.	Erythrocyte	2500 m	Y	Phosphatidylserine, ROS, intracellular calcium, CD47	N
Wang and Lin [58]	18 healthy sedentary males (22.4±1.2 years)	Crossover RCT study	Bicycle ergometer	Performed at predetermined 50% VO_2max_ for 30min under 12%, 15% and 21% O_2_. Rested in sitting position under 12%, 15% and 21% O_2_.	Lymphocyte	2733 and 4460 m	Y	CD28 and CD57	N
Mao, Fu et al. [39]	24 healthy sedentary males (22±1 years)	RCT	Bicycle ergometer	Performed at 60% WR_max_ under 15% or 21 O_2_ for 30 min/day, 5 days/week, for 5 weeks. Graded exercise test: 48 h before and after the Yintervention under 21 O_2._ Started at 0 W for 2 min, increase 20-30 W/3 min until exhaustion. Hypoxic exercise test: 24 h before and after the intervention under 12% O_2._ Warm-up at 50 W for 3 min, started 100 W for 20 min and back to 50 W for 20 min.	Erythrocyte	2733 and 4460 m	Y	CD47 and CD147	N
Wang, Chen et al. [56]	50 healthy sedentary males	RCT	Bicycle ergometer	Performed at 50% WR_max_ or 50% maximal heart rate under 21% O_2_ or 15% O_2_ for 30min/day, 5 days/week, for 4 weeks. Graded exercise test: 48 h before and after the intervention under 21 O_2._ Started at 0 W for 2min, increased 20-30 W/3 min until exhaustion.	T lymphocyte	2733m	Y	CD28 and KLRG1	Y
Risso, Turello et al. [47]	4 mountain climber (28-43 years)	Observational study	Climbing mountains	Climbing mountains from 4500 to 8160 m for 53 days.	Erythrocyte	4500 to 8160 m	N	CD47 and phosphatidylserine	N
Allsopp, Addinsall et al. [2]	20 healthy males and females (60-70 years)	RCT	Whole body exercises	Performed warm-up 5 min on a stationary bicycle. Performed leg extension, pectoral fly, standing row, and squat at 70% predetermined 1RM, 4 sets of 10 repetitions for each exercise with a 1 min rest between sets and a 2 min rest between exercises.	CD4^+^ T helper cells	3000 m	Y	CD45RA	N
Lin, Wang et al. [38]	60 healthy sedentary males	RCT	Bicycle ergometer	Performed warm-up 30% WR_max_ for 3 min. Performed at 60% WR_max_ for 30 min/day, 5 days/week, for 6 weeks. Cardiopulmonary exercise test: 4 days before and after the intervention. Performed warm-up at 0 W for 2 min. Increase 30 W/3 min until exhaustion. Hypoxic exercise test: 48 h before and after the intervention under 12% O_2._ Warm-up at 30% WR_max_ for 5 min, started at 60% WR_max_ for 30 min and 5 min cool-down at 0% WR_max_.	Erythrocyte	2733 and 4460 m	Y	CD47 and CD147	N
Wang and Wu [60]	16 healthy sedentary males (24.2±1.2 years)	Crossover RCT study	Bicycle ergometer	Performed at predetermined 50% VO_2max_ for 30min under 12%, 15% and 21% O_2_. Rested in sitting position under 12%, 15% and 21% O_2_.	Natural killer cells	2733 and 4460 m	Y	CD28 and CD57	N
Tsai, Chang et al. [50]	60 healthy sedentary males	RCT	Bicycle ergometer	Performed 80% of VO2_max_ interspersed with 3min active recovery at 40% of VO2_max_ for 30min/day, 5 days a week, for 6 weeks. Performed 60% of VO2_max_ for 30min/day, 5 days a week, for 6 weeks. Graded exercise test: 4 days before and after the intervention. Hypoxic exercise test: 24 h before and after the intervention.	Lymphocyte	4460 m	Y	CD28 and CD57	N
Allsopp, Addinsall et al. (2024a, b	20 healthy males and females (60-70 years)	RCT	Whole body exercises	Participants performed two resistance training sessions per week for eight weeks. The session consisted of four whole-body exercises performed at 70% of each participant’s predicted 1-repetition maximum; leg extension, pectoral fly, standing row and squat. Participants performed 4 sets of 10 repetitions of each exercise with a 1-minute rest between sets and a 2-minute rest between exercises.	CD 8^+^ T cells	3000 m	Y	CD45RA	Y

*SCs* Senescent cells, *ROS* Reactive oxygen species, *RCT* Randomized controlled trial, *PR* Pedal rate, *VO2*_*max*_ Maximal oxygen consumption, *WR*_*max*_ Maximal work rate, *HR*_*max*_ Maximal heart rate, *KLRG1* the killer cell lectin-like receptor G1

^a^Indicated all participants in the experiment were exposed to normobaric or hypobaric environment

**Table 3:** Summary of the effect of hypoxic exercise on sedentary individuals

Author (Year of publication)	Studied population	Exercise	Altitude	Use of normobaric hypoxia chambers^a^	Retard SCs	Effects of Hypoxic Exercise
Wang and Lin [58]	18 healthy males (22.4±1.2 years)	Bicycle ergometer	2733 and 4460 m	Y	N	12%O_2_ ME results in a greater mobilization of senescent/activated lymphocytes into the bloodstream compared to that of 15%O_2_ ME. 12%O_2_ ME leads to reduced lymphocyte antioxidant levels, thereby enhancing H_2_O_2_-induced programmed death of lymphocytes through activation of mitochondria and death receptor-mediated apoptotic pathways.
Mao, Fu et al. [39]	24 healthy males (22±1 years)	Bicycle ergometer	4460 and 2733 m	Y	N	HE downregulates erythrocyte CD47 and CD147 expression, while simultaneously promoting erythematic response to oxidative stress. HE intervention attenuates the extent of erythrocyte deformability and dehydration regulated by the Gardos channel.
Wang, Chen et al. [56]	50 healthy males	Bicycle ergometer	2733m	Y	Y	Hypoxic-absolute exercise for 4 weeks improves the aerobic fitness of the subject by enhancing pulmonary ventilation and tissue O_2_ utilization. Hypoxic exercise regimen suppresses replicative senescence of T-lymphocytes, inducing a shift towards Th1 cytokine dominance in circulation.
Lin, Wang et al. [38]	60 healthy males	Bicycle ergometer	2733 m	Y	N	An acute bout of exercise in 12%O_2_ enhanced erythrocyte aggregation and facilitated erythrocyte senescence. HE was superior to normoxic exercise training in ameliorating cardiopulmonary capacity. HE led to advanced and deteriorated rheological aggregation. Chronic hypoxic exercise may offer preconditioning acclimatization for patients with hypoxia-related diseases.
Wang and Wu [60]	16 healthy males (24.2±1.2 years)	Bicycle ergometer	2733 and 4460 m	Y	N	Both 12% and 15% O_2_ ME increased the percentages of CD62L^-^ and CD11a^+^ NKs in the bloodstream. These NKs mobilized by hypoxic ME, then rapidly left the peripheral blood compartment during the 2-hour recovery period. 12%O_2_ ME promotes NK cytotoxicity by mobilizing the replicative senescent/inhibitory NKs (CD57^+^/CD28^-^/KLRG1^+^) into the bloodstream.
Tsai, Chang et al. [50]	60 healthy males	Bicycle ergometer	4460 m	Y	N	HE increased the mobilization of senescent (CD57^+^/CD28^-^) lymphocytes into the blood. HE decreased the ATP-linked OCR, the reserve capacity of OCR, and the activity of citrate synthase in the mitochondria. HE lowered the mitochondrial membrane potential and elevated the matrix oxidant burden of lymphocytes.

*SCs* Senescent cells, *ROS* Reactive oxygen species, *ME* Moderate-intensity exercise, *HE* Hypoxic exercise training, *NK* Natural killer cell, *OCR* O_2_ consumption rate

^a^Indicated all participants in the experiment were exposed to normobaric or hypobaric environment

Regarding the physical exercise intervention, most studies (72.7%, *n*=8) utilized cycling as the exercise modality. Two articles used whole-body exercises, including leg extension, pectoral fly, standing row and squat. One study employed mountain climbing as the exercise intervention.

**Fig. 2:**
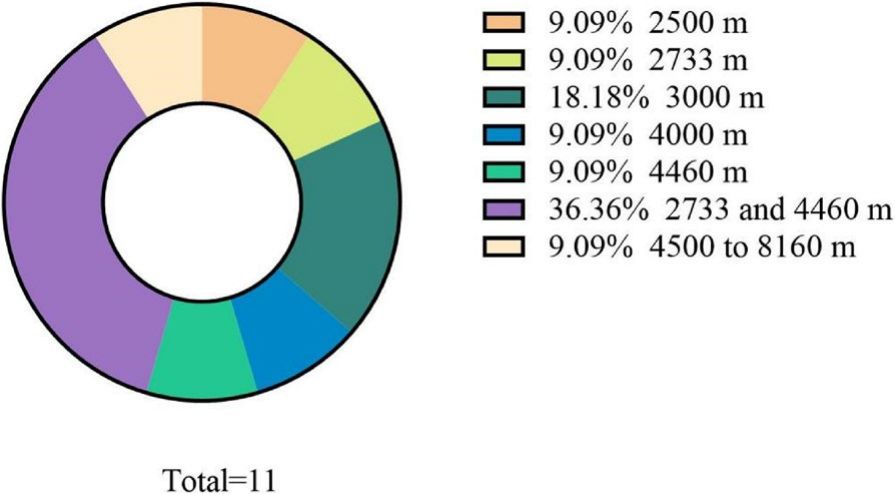
The proportion of usage at different altitudes

Regarding the approaches for creating a hypoxic environment, 10 studies (90.9%) utilized normobaric hypoxia cambers to mimic normobaric hypoxia conditions, and one study engaged participants in physical exercises in hyperbaric hypoxia setting at high-altitude locations (4500 to 8160). Within the scope of this review, the altitudes that studies attempted to replicate or reach exhibited a wide range, spanning from 2500 to 8160 m. Among the selected papers, 36.4% (n=4) opted to utilize hypoxia chambers to mimic altitudes of 2733 and 4460 m (Figure 2).

Among the 11 articles, most studies specifically investigated the effect of hypoxic exercise either on lymphocytes (54.5%, n=6) or erythrocytes (36.4%, n=4). However, various senescence markers were used to examine the cellular senescence. Articles that investigated lymphocytes generally used KLRG1, CD28, CD57, and CD45RA. Regarding erythrocytes, researchers utilized CD47, CD147, phosphatidylserine, reactive oxygen species (ROS) and intracellular calcium as senescence markers (Table 3).

In terms of the impact of hypoxic exercise on cellular senescence, three articles highlighted that hypoxic exercise could effectively retard senescence in T lymphocytes [1, 61] and circulating endothelial progenitor cells [27]. Nevertheless, eight articles indicated that hypoxic exercise did not substantially affect delayed cellular senescence across different cell types, including erythrocytes [11, 38, 39, 47], lymphocytes [50, 58], T lymphocytes [2] and natural killer cells [60].

## Discussion

### Cellular senescence in tissues

This review examined the potential anti-aging effects of hypoxic exercise on senescent cells. Three kinds of tissues were examined following the intervention of hypoxic exercise: endothelial progenitor cell, erythrocyte, and lymphocyte. The findings indicated that hypoxic exercise did not have a significant impact on retarding cellular senescence in erythrocytes. However, it showed positive effects in delaying the senescence of circulating endothelial progenitor cells and increasing their lifespan. Its effects on lymphocytes were inconclusive. Debate regarding the effectiveness of hypoxic exercise inducing an anti-aging effect on lymphocytes has, indeed, been ongoing. The available research on outcomes and conditions related to the anti-aging effect of hypoxic exercise on senescent lymphocytes is currently less definitive. Additional studies are required to further investigate and elucidate the potential anti-aging properties of hypoxic exercise with a focus on senescent cells Table 4.

**Table 4:** Summary of markers of senescent cells investigated in studies

Tissue	Number of articles (n)	Ratio (%)	Marker of SCs (the number of articles used)
Circulating endothelial progenitor cells	1	9.1	SA-β-gal (*n*=1)
Erythrocyte	4	36.4	CD47 (*n*=4) Phosphatidylserine (*n*=2) CD147 (*n*=2) ROS (*n*=1) Intracellular calcium (*n*=1)
Lymphocyte	6	54.5	CD28 (*n*=4) CD57 (*n*=3) CD45RA (*n*=2) KLRG1 (*n*=1)

*ROS* Reactive oxygen species

Senescent cells have been found to play a multifaceted role in aging, tumor development, and chronic diseases [12, 14, 41, 45, 54, 63]. Owing to the heterogeneity of senescent cells and the limited specificity of the markers, multiple techniques have been used to identify senescent cells [29], including secretory phenotype (IL-6 and ROS), cell cycle arrest (p16 and p21) [5], and senescence-associated beta-galactosidase (SA-β-Gal) [28]. In this review, Harmsen et al. investigated endothelial progenitor cells using SA-β-Gal as a marker, while Carin et al. utilized ROS to detect markers of senescence. CD57 has been recognized as a marker of replicative senescence in human lymphocytes [9], whereas CD28 plays a crucial role as a co-stimulatory molecule in the activation and proliferation of naive lymphocytes [21, 52]. Among the 6 articles that examined the impact of hypoxic exercise on lymphocytes, 4 employed CD28 as markers to assess senescent cells.

Of note, three of these articles indicated that hypoxic exercise can increase the mobilization of senescent lymphocytes into the bloodstream, but it does not retard cellular senescence in lymphocytes [50, 58, 60]. As a marker of replicative senescence in human T-lymphocytes, KLRG1 was used to demonstrate that hypoxic exercise can suppress replicative senescence of T-lymphocyte [6, 56]. Similarly, Allsopp et al. employed CD45RA as a marker of senescence and suggested that hypoxic exercise can cause a reduction in senescent CD8^+^ T cells. This phenomenon may be attributed to the ability of exercise to induce apoptosis and diminish senescent T cells by recruiting them into peripheral tissues [22]. Considering that the T cell pool is likely capped in the body, apoptosis of senescent T cells may create opportunities for the expansion of naïve T cells, thereby mitigating T cell senescence [51].

CD47 is a surface glycoprotein exposed on virtually all cells including erythrocytes. A substantial body of evidence indicated that CD47 is involved in the negative regulation of erythrocyte phagocytosis [18, 42, 43]. Furthermore, under normal circumstances, phosphatidylserine is confined to the inner leaflet of cell membranes. However, in the case of abnormal or apoptotic cells, it becomes exposed, acting as a signal to macrophages for ingestion. As a result, phosphatidylserine plays a significant role in positively regulating erythrocyte phagocytosis [8, 44]. This review encompasses four articles that consistently utilized CD47 as a critical cellular senescence marker for erythrocytes, and two of these studies specifically chose to focus on measuring phosphatidylserine as well. However, all studies indicated that hypoxic does not retard senescent cells in erythrocytes.

These findings suggest that hypoxic exercise does exhibit the capacity to retard cellular senescence, particularly in circulating endothelial progenitor cells and lymphocytes. However, due to the heterogeneity of senescent cells and the limited specificity of the markers, future investigations should adopt a combination of various techniques to provide a more comprehensive understanding of the effect of hypoxic exercise on cellular senescence [29]. This may involve assessing senescent pathways such as p53/p21^Cip1^ and p16^INK4a^/RB [48], apoptosis resistance [13], and increased lysosomal content [34], to provide a more comprehensive understanding of the effect of hypoxic exercise on cellular senescence.

### Chronic exercise for different population

Studies included in this review implemented a range of exercise modalities under hypoxic conditions. The majority (72.7%, *n*=8) employed cycling as the exercise modality, with 6 of these studies especially targeting focused on sedentary males. Two papers focused on healthy elderly participants who performed four whole-body exercises, while another study involved mountain climbing as the exercise intervention. Compared to walking and running, cycling on a stationary bike exerts less stress on the joints, making it an ideal exercise choice for sedentary participants and older adults with lower physical activity levels. Moreover, using cycle ergometers offers researchers the advantage of adjusting the intensity of physical exercise while eliminating concerns related to variables such as temperature, humidity and oxygen during the experiment. This is why many studies selected cycling as the preferred exercise modality.

Among 6 articles examining the effect of hypoxic exercise on sedentary males, only one article concluded that hypoxic exercise could retard cellular senescence [56]. Hypoxic exercise has been shown to enhance the aerobic fitness of the subject by improving pulmonary ventilation and tissue O_2_ utilization [56]. Compared to normoxic exercise, hypoxic exercise appears to be more effective in ameliorating cardiopulmonary capacity [38]. Nevertheless, it is worth noting that all of these studies focus exclusively on sedentary males. Further research should pay more attention to investigating the effects of hypoxic exercise on sedentary female populations is warranted.

### Inflammation reduction and antioxidative effect

Ageing is accompanied by immune dysregulation termed inflammageing [25], characterized by elevated levels of pro-inflammatory markers in cells and tissues, alongside reduced levels of anti-inflammatory cytokines [24, 26, 40]. Inflammation and leukocyte dysfunction in the ageing population is closely linked to chronic morbidity, disability, frailty, and must be targeted to promote healthy ageing [23].

Kiers et al. proposed that hypoxia (peripheral saturation of 80-85%) dampens the systemic pro-inflammatory cytokine response and increases anti-inflammatory cytokines when confronted with an inflammatory challenge [31]. In this review, Allsopp et al. suggested that hypoxic exercise does not affect inflammatory cytokines [1, 2]. On the contrary, Wang et al. indicated that hypoxic exercise can suppress pro-inflammatory cytokines [56]. They also suggest that conducting hypoxic exercise under 15% O_2_ instead of 12% O_2_, could enhance aerobic capacity while minimizing the risk of inducing inflammatory responses [38].

During severe hypoxia, blood undergoes oxidative stress [46, 59], and ROS facilitates rapid microvascular inflammation [62]. Excessive exposure to oxidative stress can lead to replicative senescence and apoptosis of immune cells [33, 53], thereby increasing the risk of infectious diseases and autoimmune disorders. Findings demonstrate that exercise under 12% O_2_ can enhance oxidative stress, and diminish lymphocyte antioxidative capacity, but cellular redox statuses are unchanged under 15% O_2_ [53]. In the meantime, exercise under 15% O_2_ conditions can depress oxidative stress and reduce immune dysfunction by retarding T-lymphocyte senescence [56]. Furthermore, Wang et al. also propose that exercise with/without hypoxic exposure effectively alleviates lymphocyte apoptosis induced by oxidative stress following strenuous exercise [57]. The findings from this section suggest that 15% O_2_ may be suitable for hypoxic exercise, minimizing the risk of inducing inflammatory responses and depressing oxidative stress.

## Conclusion and future work

The finding of this narrative review provides compelling evidence suggesting that hypoxic exercise has the potential to retard cellular senescence in specific cell types. Nevertheless, the research exhibited clear limitations that can not be ignored. First, this narrative review was restricted to articles published in English articles and despite a systematic search of four representative databases in the field, the number of available studies was limited. Second, there was little consensus on cellular senescence markers of various cell types, potentially leading to contradictory findings and conclusions. It is also noteworthy that most studies in this field have primarily focused on using healthy participants as experimental subjects. Limited research has been conducted on female populations and individuals with various disease states. The identified limitations should be carefully considered in the design of future research to improve its overall quality. Standardizations on the type of hypoxic exercise and marker of cellular senescence are necessary. More attention should be given to female populations and individuals with different disease states. Lastly, researchers should investigate the most effective form and dosage of exercise and the underlying cellular mechanisms.

## Data Availability

The original contributions presented in the study are included in the article/Supplementary material, further inquiries can be directed to the corresponding author.
